# High-Performance SiPM Detection Module for Ultra-Fast Time-Resolved Measurements

**DOI:** 10.3390/s26103072

**Published:** 2026-05-13

**Authors:** Gennaro Fratta, Piergiorgio Daniele, Ivan Labanca, Michele Penna, Giulia Acconcia, Alberto Gola, Ivan Rech

**Affiliations:** 1 Department of Electronics, Information and Bioengineering (DEIB) of Politecnico di Milano, 20133 Milan, Italy; piergiorgio.daniele@polimi.it (P.D.); ivangiuseppe.labanca@polimi.it (I.L.); giulia.acconcia@polimi.it (G.A.); ivan.rech@polimi.it (I.R.); 2 Fondazione Bruno Kessler (FBK), 38122 Trento, Italy; mpenna@fbk.eu (M.P.); gola@fbk.eu (A.G.)

**Keywords:** analog front-end electronics, correction algorithm, dead time, pile-up distortion, time-correlated single-photon counting, time-resolved fluorescence measurements, timing jitter, silicon photomultiplier

## Abstract

Today, the rapid progress in non-invasive light–matter interaction analysis is transforming the landscape of biomedical and life sciences driven by low-intensity light detection technologies. As the complexity of photonic applications continues to grow, the importance of single-photon detection techniques becomes pivotal. Among them, Time-Correlated Single-Photon Counting (TCSPC) has become the gold standard for precise, time-resolved reconstruction of rapid and faint optical signals. However, TCSPC has long been constrained by pile-up distortion, which worsens with increasing acquisition speed, typically limiting it to 5% of the excitation frequency. To overcome the operational constraints of conventional implementations, a novel TCSPC acquisition methodology has been introduced, independent of photodetector dead time, excitation intensity, and prior optical signal knowledge, still enabling distortion-free reconstruction of the measured light profiles. In this context, the development of single-photon detectors with short dead time and low timing jitter becomes crucial. This work presents a single-photon detection module based on a Silicon Photomultiplier, which delivers 750 ps FWHM output pulses with a 33.5 ps RMS IRF. Its performance is showcased through fluorescence measurements employing the constraint-free TCSPC methodology, achieving a photon count rate up to 166% of the excitation frequency with a minimal lifetime estimation error of just −1.46%.

## 1. Introduction

In recent years, biomedical and life sciences have been significantly revolutionized by the rapid advancement of low-intensity light detection techniques in non-invasive light–matter interaction analysis. As the complexity of photonic applications continues to grow, the role of single-photon detection techniques becomes crucial. Among them, Time-Correlated Single-Photon Counting (TCSPC) represents a powerful option considering its ability to reconstruct weak, rapidly changing optical signals with exceptional sensitivity and timing resolution, far surpassing traditional analog approaches [1,2]. The operating principle of TCSPC involves the use of a pulsed laser source to illuminate the target, which results in the emission or reflection of photons. These photons are detected by single-photon detectors, and their arrival times are measured and digitized with picosecond resolution [3,4]. Once a statistically significant number of events is collected, the stored data are processed to reconstruct the waveform of interest [5]. In particular, TCSPC has been widely adopted in applications such as Fluorescence Lifetime Imaging Microscopy (FLIM) [6,7,8], single-molecule fluorescence analysis [9], and Time-of-Flight (ToF) measurements, both in free-space and harsh environments [10,11]. Nevertheless, TCSPC has historically been limited by its inability to achieve ultra-fast data acquisition, a fundamental requirement for real-time analysis. In fact, classic TCSPC theory dictates that the maximum photon count rate must be limited to a small fraction of the excitation frequency (typically ≈5%) [1,12], thus hindering its application in high-speed scenarios that require an extremely high photon detection rate [13]. Several approaches have been reported in the literature to mitigate this limitation, including statistical correction algorithms [14,15]. However, these methods often require extensive post-processing and prior knowledge of the expected optical signal, easily preventing their applicability to real-time and unpredictable scenarios. Recently, a novel TCSPC acquisition methodology was introduced to overcome the intrinsic operational constraints of conventional approaches, enabling distortion-free reconstruction of photon arrival statistics [16]. The proposed approach is independent of the dead time of the detection system, excitation intensity, and any a priori knowledge of the optical signal under investigation, redefining the limits of TCSPC operation and extending its applicability to regimes that were previously inaccessible under standard counting assumptions. In particular, this methodology has already been experimentally validated, reaching a photon count rate of 60% of the excitation frequency using a customized commercial module based on a Single-Photon Avalanche Diode (SPAD) [17], and up to 121% when employing a Hybrid Photodetector (HPD) [18]. In this scenario, there is a growing interest in improving single-photon detector performance to achieve ultra-fast timing measurements. In fact, single-photon-sensitive devices, such as SPADs [19,20,21], Silicon Photomultipliers (SiPMs) [22,23], and HPDs [24], along with their corresponding front-end electronics, are responsible for transducing impinging light into processable electrical signals. Thus, selecting the most suitable single-photon detector for each specific application is fundamental, as it directly impacts the overall performance of the measurements.

Among the different types of single-photon detectors, SiPMs can offer exceptional detection efficiency, excellent timing resolution, and scalability, all within a compact, low-power, and cost-effective design [25,26]. SiPMs integrate an array of hundreds or thousands of SPAD micro-cells on a single silicon substrate, all connected in parallel [27,28,29,30,31]. Compared to a single SPAD, where the limited active area constrains photon collection efficiency, often necessitating complex optical focusing systems, SiPMs provide a significantly larger effective detection area, enhancing sensitivity and photon detection capabilities [32]. Furthermore, their parallel architecture simplifies the design of the readout electronics in contrast to SPAD arrays, where each pixel requires an individual readout channel [33]. Compared to HPDs, SiPMs can potentially achieve higher maximum count rates and their solid-state nature ensures robustness against external electromagnetic interference and mechanical stress, making them ideal for miniaturized optical systems that demand portability and durability. In addition, SiPMs offer excellent scalability, allowing the integration of multiple photodetectors into arrays. Last but not least, modern SiPMs benefit from advanced fabrication techniques that result in low afterpulsing probability, crosstalk, and dark counts, effectively minimizing false-photon events that could otherwise compromise data integrity [26,34].

Given the challenges intrinsically introduced by the distributed micro-cell architecture of the sensor, this work introduces a SiPM-based single-photon detection module, whose front-end is specifically optimized for both high-precision photon timing extraction and ultra-short dead time. By leveraging the aforementioned constraint-free TCSPC methodology, we demonstrate groundbreaking capabilities in ultra-fast fluorescence measurements, achieving a photon count rate as high as 166% of the excitation frequency with minimal distortion. Beyond ensuring exceptional accuracy in lifetime estimation through the pile-up correction strategy, we also show a high-fidelity reconstruction of the measured optical signals employing a recently reported algorithm that compensates for second-order distortions visible at high-photon fluxes, preserving data integrity [18].

The manuscript is structured as follows: Section 2 provides a comprehensive analysis of the SiPM device, detailing its physical and electrical parameters and front-end design requirements. Section 3 focuses on the architecture of the detection module, highlighting the front-end electronics design. Section 4 details the component selection strategy to minimize timing jitter and dead time, and compares the experimentally determined figures of merit with predictions from simulations. Section 5 briefly reviews the main principles of the constraint-free pile-up correction methodology and the algorithm to compensate residual distortion effects, and presents the experimental validation of the detection module through high-photon-flux fluorescence measurements. Finally, Section 6 concludes the manuscript, summarizing the key contributions and highlighting potential directions for future research to further advance the field.

## 2. SiPM Parameters and Front-End Design Requirements

This section presents the physical parameters of the SiPM employed in this study and outlines the simulation framework adopted to optimize the front-end readout architecture.

### 2.1. Physical Parameters

The SiPM was designed and manufactured by Fondazione Bruno Kessler (FBK) and belongs to the NUV-Sensitive technology family, particularly NUV-HD [32,34]. Briefly, due to the reduced cell pitch and the resulting high micro-cell density, this technology achieves an enhanced dynamic range and a reduced recovery time, while maintaining low correlated noise [32,34], which is particularly advantageous in the high photon-flux TCSPC regime addressed in this work, especially when combined with the proposed pile-up correction strategy reported in Section 5. Moreover, these characteristics enable reliable operation at high overvoltage, thereby increasing the photon detection efficiency without degrading the Signal-to-Noise Ratio (SNR) [32,34], which is significantly relevant in application scenarios where the excitation intensity is constrained by sample-related limitations. The selected sensor is characterized by an active area, $A_{\mathrm{SiPM}}$, equal to 1mm×1mm, with a micro-cell pitch roughly equal to 15μm and a fill factor of 63.6%. At an operating temperature of 10 °C, the device exhibits a dark count rate of ≈10 kcps. The breakdown voltage, $V_{\mathrm{BD}}$, is ≈32 V, and, in this study, the device was biased with an overvoltage, $V_{\mathrm{OV}}$, equal to 10V, resulting in a bias voltage of 42V.

### 2.2. Electrical Parameters and Simulation Model

**Figure 1:**
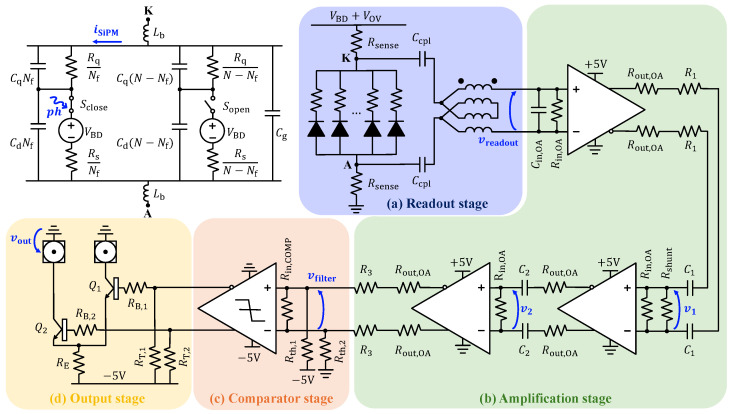
Front-end circuit schematic, with signal processing chain proceeding from top-left to bottom-left in a clockwise direction. The top-left frame highlights the circuit model of the sensor. The blue frame (**a**) illustrates the fully differential readout stage, the green frame (**b**) depicts the three-stage amplification path, the orange frame (**c**) shows the comparator stage, and the yellow frame (**d**) represents the output stage.

The SiPM circuit model—previously reported in [25,26,35,36,37,38,39,40,41]—is shown within the top-left frame in Figure 1. It represents the equivalent configuration of all micro-cells, *N*, distinguishing between those fired upon photon absorption, $N_{f}$, and those remaining in a quiescent state, waiting for activation, $N-N_{f}$. Each micro-cell consists of a SPAD in series with its passive quenching network. The SPAD is electrically modeled as the parallel of a depletion layer capacitance, $C_{d}$, with the series of a controlled switch, *S*; a voltage source equal to $V_{\mathrm{BD}}$; and a resistance, $R_{s}$ [25,26,37,38,39,40,41]. The quenching network is represented by a quenching resistor, $R_{q}$, in parallel with a parasitic capacitance, $C_{q}$. Since all micro-cells are connected in parallel, the active network, as well as the inactive network, behaves as a parallel combination of each contributing element. The model, conceived as two interacting macro-blocks, is sufficient for the intended analysis since there is no need to distinguish which specific micro-cell is triggered. Finally, the simulation model accounts for the grid capacitance, $C_{g}$, and the inductive effects of the bonding wires required to connect the sensor to the package, as well as those of the package itself, by incorporating a parasitic bonding inductance, $L_{b}$, at each terminal [25,42].

To investigate the electrical response of the device and support the design of the front-end architecture, circuit-level simulations were performed using LTspice (Analog Devices, Inc., Wilmington, MA, USA). The values of the passive internal components were provided by the manufacturer. The device comprises 4830 micro-cells. *S* is driven by the logical output of a two-input OR gate, which combines a short excitation pulse with an arbitrary width of a few tens of picoseconds (i.e., the impinging photon) and a digital signal derived from the SPAD current, accordingly with the approach reported in [38]. Specifically, the latter monitors the quenching state: when the current drops below 100μA and it is no longer sufficient to sustain the avalanche process (considering the current asymptotic minimum value $\approx \frac{V_{\mathrm{OV}}}{R_{q}}=20\;\mu A$), the signal is forced low and *S* opens; otherwise, the signal is held high, keeping the switch closed [25,26,37,38,39,40,41,43]. The parasitic inductance associated with the bonding wires and package interconnects was estimated by modeling the differential connection between each SiPM terminal and the Printed Circuit Board (PCB) as a series inductive path carrying equal currents in opposite directions and by accounting for both self- and mutual-inductance contributions. The SiPM is housed in a 12-pin TO package; therefore, the contribution of the package was evaluated by approximating each connection pillar as a cylindrical conductor with a length of ≈7 mm and a radius of ≈0.35 mm, separated by 2.5mm. Also taking into account the contribution of the bonding wire, $L_{b}$ was conservatively estimated to be approximately 4nH.

The complete set of component parameters used in the simulation model is summarized in Table 1. All values show good agreement with the electrical behavior of the SiPM experimentally observed.

### 2.3. Front-End Design Requirements

Optimization of the front-end electronics in SiPM-based detection systems is intrinsically challenging due to the distributed micro-cell architecture of the sensor and its complex, strongly coupled electrical dynamics [44]. In ultra-fast time-resolved applications, the front-end must therefore be engineered with particular care to overcome the classical trade-off between timing precision and dead time. A conventional analog front-end consists of a current-sensing readout stage, a broadband amplification chain and a threshold discriminator.

Concerning timing precision, under small-signal conditions, it can be approximated as the ratio between the total voltage noise and the signal slew rate at the discriminator input at the threshold crossing [26,42,44,45,46,47,48], namely

(1) $$\sigma_{t}=\frac{\sigma_{v,\mathrm{noise}}}{{\left.\frac{dv}{dt}\right|}_{\mathrm{threshold}}}.$$

Regarding dead time, unlike single-junction detectors, SiPMs do not exhibit a sensor-level dead time owing to their parallel micro-cell topology. Although each fired micro-cell requires several nanoseconds to recharge through its quenching network, inactive micro-cells remain fully sensitive and capable of detecting subsequent photons. Consequently, the dominant contribution to the effective system dead time originates from the front-end electronics rather than from the sensor itself. In this work, the system dead time is defined as the interval following a detection event during which the electronic chain is unable to register subsequent photons, and it is related to the time required for the output pulse to return below a defined low-threshold level. Therefore, both timing jitter and dead time are governed by the analog front-end architecture, particularly the band-pass characteristics imposed by the electrical path preceding the discrimination stage [25,46].

The distributed micro-cell topology of the sensor inherently hinders the efficient extraction of the high-frequency components of the single-cell avalanche current [49], thereby limiting both the attainable timing precision and the minimum achievable dead time. When the system bandwidth falls below the cut-off frequency determined by the bi-exponential impulse response of the single cell [39], the rising-edge slew rate progressively degrades. Conversely, under the assumption of a white noise spectrum, extending the bandwidth beyond this limit does not further increase the slew rate at the discriminator input but instead enhances the integrated noise power. The opposite behaviors imply the existence of an optimal front-end bandwidth that minimizes timing uncertainty [42,50] with a beneficial effect on the effective dead time. In practice, however, this theoretical optimum cannot be fully exploited, as the overall system response is constrained by the parasitic inductances of the bonding wire and package, the input network impedance, and the finite gain–bandwidth product of the amplification stages.

Complementary to the low-pass constraints, the front-end must incorporate an appropriate high-pass action if short dead times in high count-rate operation are required. In a SiPM, simultaneous photon absorption in different micro-cells results in the superposition of independent avalanche pulses. To temporally resolve proximate photon events, the front-end must therefore suppress the long, slowly recovery tail following each detection event, while preserving the fast-rising edge of the micro-cell avalanche current. This would simultaneously suppress low-frequency noise, ultimately reducing timing jitter. However, excessively small coupling capacitances introduce signal-partitioning effects that can significantly attenuate the useful signal component and may also filter out high-frequency content, degrading the slew rate and thus the timing performance. Moreover, it typically produces an undershoot that distorts the pulse shape and potentially introduce baseline fluctuations [25,45,46], compromising the temporal accuracy of the timestamping process—a key metric on par with timing jitter and dead time, as discussed in Section 5.

## 3. Front-End Electronics Design

The front-end architecture is illustrated in Figure 1 and comprises four main functional blocks: a fully differential readout stage (blue frame in Figure 1a), a three-stage amplification path (green frame in Figure 1b), a comparator stage (orange frame in Figure 1c), and an output stage (yellow frame in Figure 1d). It is worth emphasizing that this section describes the implementation of the front-end architecture, whereas the selection and optimization of each component are rigorously addressed in Section 4.

### 3.1. Fully Differential Signal Readout

The readout stage is highlighted in the blue frame of Figure 1a. Two sensing resistors, $R_{\mathrm{sense}}=39\;\Omega$, are placed at both SiPM terminals: one between the anode (node A) and ground, and the other between the cathode (node K) and the bias voltage. The differential approach was preferred to a simpler single-ended solution to improve common-mode noise rejection and reduce susceptibility to ground and power-supply bounce, as well as to external electromagnetic interference [44]. In addition, this topology compensates for the impact of parasitic inductance associated with the bonding wires and package interconnects. Two identical coupling capacitors, $C_{\mathrm{cpl}}=820\;\mathrm{pF}$, are placed at each terminal mainly to block DC components while allowing the fast component of the avalanche current to pass through for subsequent processing. The high-pass-filtered differential voltage signal is then fed into the first amplification stage, at whose input is placed the parallel of the internal input resistance of the amplifier, $R_{\mathrm{in},\mathrm{OA}}=100\;\Omega$, and an external capacitor, $C_{\mathrm{in},\mathrm{OA}}=1.4\;\mathrm{pF}$. In particular, if the SiPM was directly connected to the amplifier input terminals, the avalanche current pulse would preferentially flow through the series-sensing resistors rather than into the amplifier input, since their combined equivalent high-frequency impedance would be lower than $R_{\mathrm{in},\mathrm{OA}}$. As a consequence, the current injected into the active input node would be reduced, leading to an SNR degradation. Therefore, to mitigate this effect and maximize signal transfer, a wide-band RF transformer was employed, following the approach outlined in [51] and subsequently adopted in [48,52,53]. The component (TC4-19G2, Mini-Circuits, Brooklyn, NY, USA) has a turn ratio $n_{\mathrm{turn}}=2$ and operates over a frequency range from 10MHz to 1.9GHz. Due to the impedance transformation property, the transformer reduces the input impedance reflected to the primary by a factor $n_{\mathrm{turn}}^{2}=4$, thereby lowering the effective load seen by the SiPM current pulse and improving current transfer into the front-end stage. Compared with the use of an external resistor at the amplifier input—which would provide a similar reduction in equivalent input impedance—the transformer-based solution simultaneously doubles the differential voltage at the amplifier input, resulting in an improved SNR. To accurately capture the frequency-dependent behavior of the transformer and its resulting contribution to the overall SNR, the transformer was modeled as an equivalent parallel LC network characterized by an inductance $L_{\mathrm{TR}}=400\;\mathrm{nH}$ and a capacitance $C_{\mathrm{TR}}=3.5\;\mathrm{pF}$ connected in parallel with the transformer-scaled input network of the operational amplifier. This equivalent network replaces the transformer illustrated in the blue-highlighted region of Figure 1a and is considered connected in parallel with the operational amplifier input stage. Finally, it is essential to account for the impact of the equivalent high-frequency capacitance of the sensor, $C_{\mathrm{SiPM}}$, which can be considered as replacing the sensor model within the readout stage of Figure 1a. Based on the device parameters reported in Table 1, this capacitance can be expressed as

(2) $$\begin{array}{cc} C_{\mathrm{SiPM}} & \approx \left(N-N_{f}\right)\cdot \frac{C_{d}C_{q}}{C_{d}+C_{q}}+C_{g}=17.88\;\mathrm{pF}. \end{array}$$

The input network can be modeled as a band-pass filter, whose transfer function is

(3) $$\begin{array}{ll} \frac{v_{\mathrm{readout}}}{i_{\mathrm{SiPM}}}\left(s\right)= & -\frac{2\;n_{\mathrm{turn}}\;\nu \;s^{2}}{1+\delta_{1}s+\delta_{2}s^{2}+\delta_{3}s^{3}+\delta_{4}s^{4}+\delta_{5}s^{5}},\\ \nu = & \frac{1}{2}L_{\mathrm{TR}}R_{\mathrm{sense}}C_{\mathrm{cpl}},\\ \delta_{1}= & 4\frac{L_{\mathrm{TR}}}{R_{\mathrm{in},\mathrm{OA}}}+R_{\mathrm{sense}}\left(C_{\mathrm{cpl}}+2C_{\mathrm{SiPM}}\right),\\ \delta_{2}= & 4\frac{L_{\mathrm{TR}}}{R_{\mathrm{in},\mathrm{OA}}}[\frac{1}{8}R_{\mathrm{in},\mathrm{OA}}\left(2C_{\mathrm{TR}}+8C_{\mathrm{in},\mathrm{OA}}+C_{\mathrm{cpl}}\right)+\\ & +R_{\mathrm{sense}}\left(C_{\mathrm{cpl}}+2C_{\mathrm{SiPM}}\right)]+L_{b}C_{\mathrm{cpl}},\\ \delta_{3}= & 4\frac{L_{\mathrm{TR}}}{R_{\mathrm{in},\mathrm{OA}}}[\frac{1}{8}R_{\mathrm{in},\mathrm{OA}}R_{\mathrm{sense}}(\left(2C_{\mathrm{TR}}+8C_{\mathrm{in},\mathrm{OA}}\right)C_{\mathrm{cpl}}+\\ & +2\left(2C_{\mathrm{TR}}+8C_{\mathrm{in},\mathrm{OA}}\right)C_{\mathrm{SiPM}}+2C_{\mathrm{cpl}}C_{\mathrm{SiPM}})+\\ & +2L_{b}C_{\mathrm{SiPM}}]+2L_{b}R_{\mathrm{sense}}C_{\mathrm{cpl}}C_{\mathrm{SiPM}},\\ \delta_{4}= & 8\frac{L_{\mathrm{TR}}L_{b}C_{\mathrm{SiPM}}}{R_{\mathrm{in},\mathrm{OA}}}[\frac{1}{8}R_{\mathrm{in},\mathrm{OA}}\left(2C_{\mathrm{TR}}+8C_{\mathrm{in},\mathrm{OA}}\right)+\\ & +\left(\frac{1}{8}R_{\mathrm{in},\mathrm{OA}}+R_{\mathrm{sense}}\right)C_{\mathrm{cpl}}],\\ \delta_{5}= & L_{\mathrm{TR}}L_{b}R_{\mathrm{sense}}\left(2C_{\mathrm{TR}}+8C_{\mathrm{in},\mathrm{OA}}\right)C_{\mathrm{cpl}}C_{\mathrm{SiPM}}. \end{array}$$

The overall transfer function exhibits two zeros at the origin and a pair of low-frequency complex–conjugate poles located at ≈6.1 MHz. The associated quality factor, computed from the real and imaginary components, is Q≈0.53, corresponding to a near-critically damped regime, crucial for suppressing the onset of spurious oscillatory modes. At higher frequencies, the transfer function introduces a real pole at ≈506.9 MHz and a complex–conjugate pole pair centered at ≈569.7 MHz characterized by a quality factor Q≈1.39. Although the pole pair is formally under-damped, the selected quality factor reflects a near-optimal compromise with respect to the targeted design objectives. It produces an exponentially decaying envelope with a sub-nanosecond time constant, ensuring rapid attenuation of residual oscillations and resulting in a fast, well-controlled transient response.

### 3.2. Three-Stage Amplification Stage

Additional post-readout gain is required to ensure adequate signal amplitude for reliable single-photon discrimination and downstream processing. The differential signal, $v_{\mathrm{readout}}$, is therefore processed through a three-stage amplification chain, as highlighted in the green frame of Figure 1b. Fully differential amplifiers (ADL5569, Analog Devices, Inc., Wilmington, MA, USA) are employed in each stage. The selected device provides a nominal in-band gain of 20dB and a wide −3dB bandwidth of 6.5GHz. A simulation model of the amplifier is available from the manufacturer. The effect of the amplification stage can be modeled by the following transfer function:

(4) $$\begin{array}{cc} G_{\mathrm{OA}}\left(s\right) & =\frac{10}{1+\delta s},\\ \delta & =\frac{1}{2\pi \cdot 6.5\;\mathrm{GHz}}. \end{array}$$

The differential output of the first amplifier is terminated by a resistor, $R_{1}=43\;\Omega$, placed in series with the internal output resistance of the amplifier, $R_{\mathrm{out},\mathrm{OA}}=7\;\Omega$. This arrangement yields an effective output impedance of 50Ω, thereby ensuring proper impedance matching and suppressing back-reflections along the interstage transmission path. A coupling capacitor, $C_{1}=1\;\mathrm{nF}$, is placed in series with each output to suppress potential DC offsets. At the input of the second identical differential amplifier, a shunt resistor, $R_{\mathrm{shunt}}=82\;\Omega$, is connected in parallel to $R_{\mathrm{in},\mathrm{OA}}$, effectively lowering the net termination impedance and enabling a controlled redistribution of gain across the cascaded amplification stages. The resulting transfer function introduces a zero at the origin and a pole at ≈2.2 MHz, and can be written as

(5) $$\begin{array}{cc} \frac{v_{1}}{v_{\mathrm{readout}}}\left(s\right) & =-G_{\mathrm{OA}}\left(s\right)\;\frac{\nu s}{1+\delta s},\\ \nu & =C_{1}\frac{R_{\mathrm{shunt}}\|R_{\mathrm{in},\mathrm{OA}}}{2},\\ \delta & =C_{1}\left(R_{1}+R_{\mathrm{out},\mathrm{OA}}+\frac{R_{\mathrm{shunt}}\|R_{\mathrm{in},\mathrm{OA}}}{2}\right). \end{array}$$

The second amplification stage feeds directly into a third identical amplifier. The two stages are connected using only coupling capacitors, $C_{2}=20\;\mathrm{pF}$, with beneficial effects on the high-pass filtering action, as will be discussed in Section 4. The resulting transfer function introduces a zero at the origin and a pole at ≈139.7 MHz and can be written as

(6) $$\begin{array}{cc} \frac{v_{2}}{v_{1}}\left(s\right) & =-G_{\mathrm{OA}}\left(s\right)\;\frac{\nu \;s}{1+\delta \;s},\\ \nu & =C_{2}\frac{R_{\mathrm{in},\mathrm{OA}}}{2},\\ \delta & =C_{2}\left(R_{\mathrm{out},\mathrm{OA}}+\frac{R_{\mathrm{in},\mathrm{OA}}}{2}\right). \end{array}$$

Finally, the third stage of amplification directly drives the comparator in the subsequent stage. Each differential output is loaded with a resistor, $R_{3}=43\;\Omega$, in series with $R_{\mathrm{out},\mathrm{OA}}$. Given the external termination resistor at the comparator input, $R_{\mathrm{in},\mathrm{COMP}}=100\;\Omega$, the resulting transfer function is purely resistive and can be expressed as

(7) $$\frac{v_{\mathrm{filter}}}{v_{2}}\left(s\right)=-G_{\mathrm{OA}}\left(s\right)\cdot \frac{\frac{R_{\mathrm{in},\mathrm{COMP}}}{2}}{R_{3}+R_{\mathrm{out},\mathrm{OA}}+\frac{R_{\mathrm{in},\mathrm{COMP}}}{2}}.$$

The resulting front-end transfer function, $\frac{v_{\mathrm{filter}}}{i_{\mathrm{SiPM}}}\left(s\right)$, is obtained as the cascade product of the individual stage transfer functions and exhibits a well-defined band-pass response, whose spectral profile is determined by the interplay of strategically positioned zeros and poles. It is important to emphasize that the overall transfer function describes the frequency-domain behavior of the front-end chain while accounting for the equivalent high-frequency capacitance of the sensor, $C_{\mathrm{SiPM}}$. However, it does not explicitly incorporate the impedance-partitioning effect of the avalanche current generated within an individual micro-cell among the SPAD junction impedance and the series of the quenching network and the equivalent input impedance of the readout electronics. This modeling choice was intentionally made to decouple the contribution of the front-end electronics from that of the sensor, since the intrinsic parameters of the device are technology-dependent and fixed by design. Consequently, from a system-level perspective, maximizing current extraction efficiency fundamentally requires minimizing the equivalent input impedance of the readout stage, thereby reducing current diversion toward parasitic sensor branches and enhancing the fraction of avalanche charge converted into a measurable voltage signal [39,41].

### 3.3. Comparator Stage

The comparator stage, highlighted in the orange frame of Figure 1c, converts the amplified analog signal, $v_{\mathrm{filter}}$, into a well-defined digital output, providing precise discrimination of photon events. An Emitter-Coupled Logic (ECL) differential comparator (ADCMP563, Analog Devices, Inc., Wilmington, MA, USA) was selected for its high-speed performance, supporting an equivalent input rise-time bandwidth of 1.5GHz, a maximum toggle rate up to 800MHz, and a minimum pulse width of 700ps. Moreover, according to the datasheet, it introduces a negligible timing jitter of 1psRMS. The comparator exhibits a negligible differential input capacitance of 0.75pF and features a high differential input resistance made negligible by an external termination resistor, $R_{\mathrm{in},\mathrm{COMP}}=100\;\Omega$. Photon-event discrimination is achieved by applying a threshold voltage offset of approximately 100mV to the comparator input. This value was selected to balance noise rejection and slew-rate preservation, ensuring a robust suppression of noise-induced crossings without compromising the input slew rate at the discrimination point [46]. The threshold was implemented using two resistors, $R_{\mathrm{th},1}$ and $R_{\mathrm{th},2}$, each with a value of 1.2kΩ, connected as shown in Figure 1c.

High-speed performance is further ensured through careful PCB design. Moreover, the power-supply network is rigorously conditioned with bypass capacitors placed close to all power pins, and the comparator inputs are placed close to the output of the third amplification stage to minimize signal degradation arising from parasitic inductive and capacitive effects.

### 3.4. Output Stage

The output stage, highlighted in the yellow frame of Figure 1d, is responsible for delivering a standardized, well-defined signal compatible with external event-timer systems. This functionality is implemented via a traditional ECL-to-NIM conversion stage. Proper termination of the ECL signals is achieved using two resistors, $R_{T,1}$ and $R_{T,2}$ each with a value of 330Ω, connected between the comparator outputs and the supply of −5V. The generation of NIM pulses is accomplished using two identical wide-band NPN transistors, $Q_{1}$ and $Q_{2}$ (BFR93A, NXP Semiconductors, Eindhoven, The Netherlands), selected for their 6-GHz bandwidth. The series resistors at the bases of $Q_{1}$ and $Q_{2}$, $R_{B,1}$ and $R_{B,2}$, both with a value of 33Ω, limit the base current to prevent excessive loading and protect the devices. The emitters are biased through a resistor, $R_{E}=220\;\Omega$, connected to the −5V supply. At the collector side, both $Q_{1}$ and $Q_{2}$ are directly connected to SMA output connectors.

All front-end component parameters, along with their corresponding values, are listed in Table 2. It is important to emphasize that the proposed front-end design is, in principle, broadly applicable to different SiPM technologies; however, the specific component values reported herein were optimized for the particular device investigated in this study.

## 4. Front-End Optimization Procedure

The front-end must be engineered to simultaneously maximize the fraction of avalanche currents effectively transferred to the readout chain, preserve the high-frequency spectral components responsible for precise timing discrimination, and enhance pulse separability to increase detection efficiency and improve the effectiveness of the pile-up correction strategy. Since the readout stage impedance directly governs how the avalanche current is partitioned within the micro-cell and toward the external electronics, component selection becomes the primary design lever. As anticipated in Section 3, this section explicitly motivates the selection of each component to substantiate the performance achieved.

### 4.1. Readout Component Selection

The readout stage represents the most critical block of the entire front-end chain. From a high-frequency perspective, it is well established in the literature that the achievable bandwidth is strongly influenced by the parasitic inductance associated with the bonding wires and package, $L_{b}$, as well as by the equivalent high-frequency capacitance of the sensor resulting from the parallel micro-cell architecture, $C_{\mathrm{SiPM}}$. Both performance metrics addressed in this manuscript may, in principle, benefit from an extension of the effective front-end bandwidth up to a certain limit, achievable through a substantial reduction in $L_{b}$ and/or $C_{\mathrm{SiPM}}$ [25,41,42]. Although the impact of $L_{b}$ on the proposed front-end is partially mitigated by the fully differential topology, it could be further reduced through direct chip-to-PCB bonding. In contrast, $C_{\mathrm{SiPM}}$ can be reduced by architectural segmentation (i.e., reducing the number of micro-cells) [50] or by adopting digital SiPM architectures [54,55]. However, both parameters are ultimately constrained by the implementation of the layout and the intrinsic technology of the sensor and therefore cannot be treated as tunable variables within the readout optimization framework. In contrast, the input capacitance of the operational amplifier, $C_{\mathrm{in},\mathrm{OA}}$, together with the high-frequency contribution of the transformer plays a crucial role at very high frequencies, shaping the high-frequency roll-off and contributing to the overall stability of the front-end, empirically demonstrating an improved suppression of propagated noise within the amplification chain.

From a low-frequency perspective, the coupling capacitor, $C_{\mathrm{cpl}}$, is essential to block DC components, particularly at the cathode node, while introducing a high-pass action. Aggressive high-pass filtering is avoided in the readout stage to preserve the SNR at the amplifier input, which would otherwise be degraded by small coupling capacitors. Instead, stronger high-pass filtering is applied in the final stage, where it suppresses the input-referred noise of the preceding amplification chain, reducing its contribution to timing jitter without affecting the initial current-to-voltage conversion.

The transformer-scaled equivalent input resistance, $\frac{R_{\mathrm{in},\mathrm{OA}}}{4}$, must satisfy two conflicting design requirements. On one hand, it must be sufficiently low compared to the series sensing resistances to maximize the efficiency of current extraction [25,41,44,49]; on the other hand, it must be sufficiently high to ensure adequate damping of potential resonance phenomena associated with the equivalent readout network, as described in Equation (3) [42,50]. System-level simulations identified an optimal reflected input resistance in the range of 22–28Ω. With the selected value of $\frac{100}{4}\;\Omega$, an average in-band current extraction efficiency of approximately 80% is achieved, while maintaining the minimum attainable quality factor for the high-frequency complex–conjugate pole pair. Finally, the sensing resistor, $R_{\mathrm{sense}}$, must be carefully dimensioned. At high frequency, it should not dominate the parallel combination with the effective input resistance, in order to preserve current extraction efficiency. However, being in series with the device, excessively high values would introduce significant voltage drops during avalanche events, transiently reducing the effective overvoltage. Since overvoltage stability is essential—especially at high count rates—this effect must be minimized. A value of 39Ω was selected as a balanced compromise. This design only marginally reduces the equivalent high-frequency input resistance, while yielding a quality factor close to critical damping for the low-frequency complex–conjugate pole pair. It also limits the post-peak voltage drop to ≤130 μV per pulse at nodes A and K, and it guarantees rapid overvoltage recovery.

### 4.2. Simulation Performance Metrics

**Figure 2:**
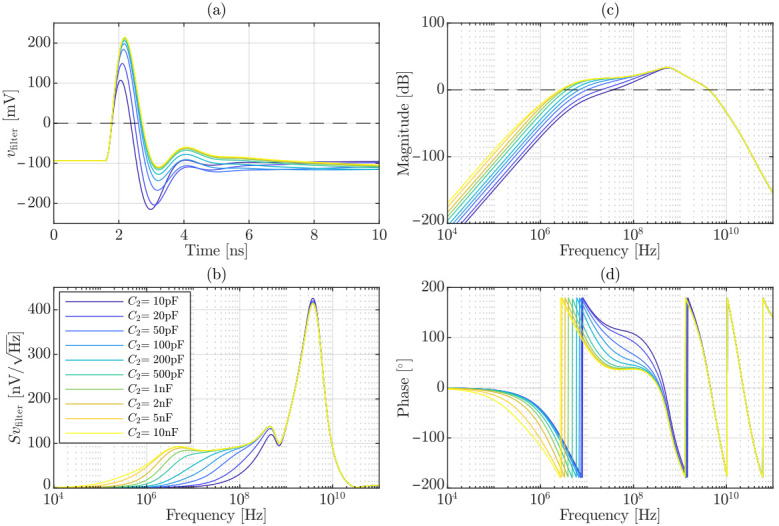
Simulated response of the complete front-end for different values of the high-pass capacitor $C_{2}$: (**a**) differential output pulse at the comparator input, $v_{\mathrm{filter}}$; (**b**) noise spectral density at the comparator input; (**c**) magnitude; and (**d**) phase Bode plots of the complete transfer function.

Having established the readout component values, the effect of the high-pass filtering must be quantitatively characterized. Since the upper bandwidth limit is dictated by non-accessible elements, the optimization analysis is therefore focused on the low-frequency shaping introduced by $C_{2}$. This study specifically evaluates its impact on the key performance metrics targeted in this work. The differential output pulse at the comparator input, $v_{\mathrm{filter}}$, together with the noise spectral density referred to the comparator input and the corresponding magnitude and phase Bode plots (referred to the current generated within a single micro-cell), are reported in Figure 2a–d, respectively. In these simulations, the avalanche event was modeled as a current generator in parallel with $C_{d}$, while the controlled switch *S* was considered open, thereby accounting for the intrinsic effect imposed by the quenching network and the parallel micro-cell architecture [35,36].

**Figure 3:**
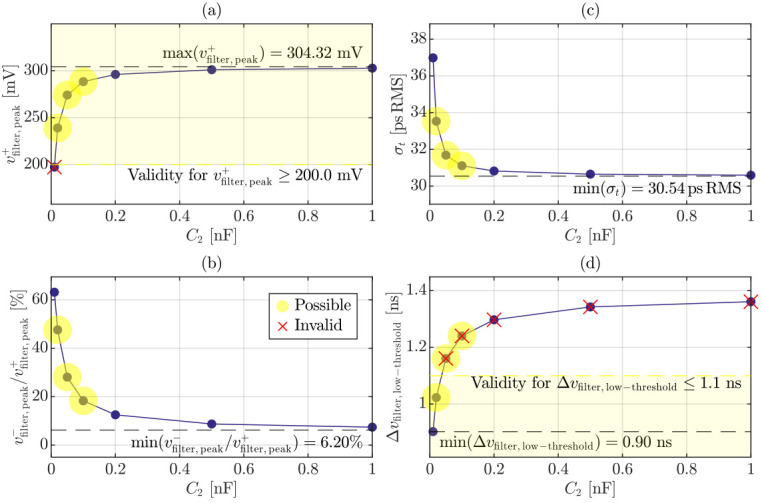
Simulated metrics as a function of the high-pass capacitor $C_{2}$ referred to $v_{\mathrm{filter}}$: (**a**) peak amplitude, $v_{\mathrm{filter},\mathrm{peak}}^{+}$; (**b**) undershoot-to-peak ratio, $v_{\mathrm{filter},\mathrm{peak}}^{-}/v_{\mathrm{filter},\mathrm{peak}}^{+}$; (**c**) RMS timing jitter, $\sigma_{t}$; and (**d**) pulse width measured at low-threshold level, $\Delta v_{\mathrm{filter},\mathrm{low}\text{-}\mathrm{threshold}}$. The shaded yellow markers identify the optimal design values located at the “knee” of the curves, while red crosses denote parameter values outside the admissible design space (transparent yellow).

Figure 3 summarizes the four adopted figures of merit: (a) pulse peak amplitude, $v_{\mathrm{filter},\mathrm{peak}}^{+}$; (b) undershoot-to-peak ratio, $v_{\mathrm{filter},\mathrm{peak}}^{-}/v_{\mathrm{filter},\mathrm{peak}}^{+}$; (c) timing jitter, $\sigma_{t}$; and (d) pulse width measured at low-threshold level, $\Delta v_{\mathrm{filter},\mathrm{low}\text{-}\mathrm{threshold}}$. In particular, $\sigma_{t}$ was evaluated according to (1) as

(8) $$\sigma_{t}=\frac{\sqrt{\int_{\begin{array}{c} \mathrm{Operating}\\ \mathrm{bandwidth} \end{array}}S_{v_{\mathrm{filter}}}^{2}\left(f\right)\;df}}{{\left.\frac{dv_{\mathrm{filter}}}{dt}\right|}_{0\;\mathrm{mV}}},$$

where the integration is performed over an operating bandwidth up to 10GHz, representing a conservative upper bound for the subsequent stage bandwidth, consistent with a worst-case optimization approach. The analysis is reported up to $C_{2}=1\;\mathrm{nF}$, beyond which all performance metrics exhibit saturation. In the component selection procedure, two primary constraints were imposed: $v_{\mathrm{filter},\mathrm{peak}}^{+}\geq 200\;\mathrm{mV}$ to guarantee a proper discrimination of the comparator and $\Delta v_{\mathrm{filter},\mathrm{low}\text{-}\mathrm{threshold}}\leq 1.1\;\mathrm{ns}$ to challenge typical dead time limitation imposed by analog architectures. The optimal design values correspond to those located at the “knee” of the saturation curves, highlighted in Figure 3 with transparent yellow markers, whereas red crosses denote values that fall outside the admissible region. Within the feasible design window (few tens of pF), a value of 20pF was selected. This choice results in the following performance metrics: $v_{\mathrm{filter},\mathrm{peak}}^{+}\approx 239\;\mathrm{mV}$, $\frac{v_{\mathrm{filter},\mathrm{peak}}^{-}}{v_{\mathrm{filter},\mathrm{peak}}^{+}}\approx 47.6\%$, $\sigma_{t}\approx 33.5\;\mathrm{ps}\;\mathrm{RMS}$ and $\Delta v_{\mathrm{filter},\mathrm{low}\text{-}\mathrm{threshold}}\approx 1\;\mathrm{ns}$.

### 4.3. Module Performance Metrics

**Figure 4:**
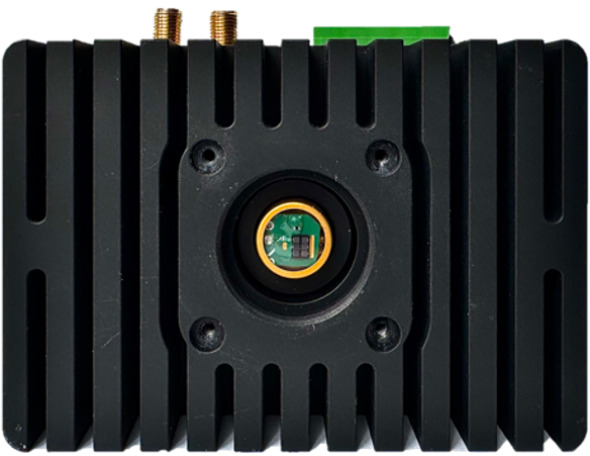
Photograph of the custom detection module.

A photograph of the fully assembled module is shown in Figure 4. The size of the system is 10cm×7cm×3.7cm and is housed in a metal enclosure, with the aim of shielding the sensitive internal circuitry from external electromagnetic interference. As introduced in Section 2, the SiPM is housed in a 12-pin TO package that includes a Negative Temperature Coefficient (NTC) thermistor (ERTJ Series, Panasonic Industry Co., Ltd., Tokyo, Japan), enabling closed-loop temperature regulation through an external Thermoelectric Cooler (TEC) unit (MS Series, Tark Thermal Solutions, Inc., Rosenheim, Germany). The module consumes approximately 1.8W.

**Figure 5:**
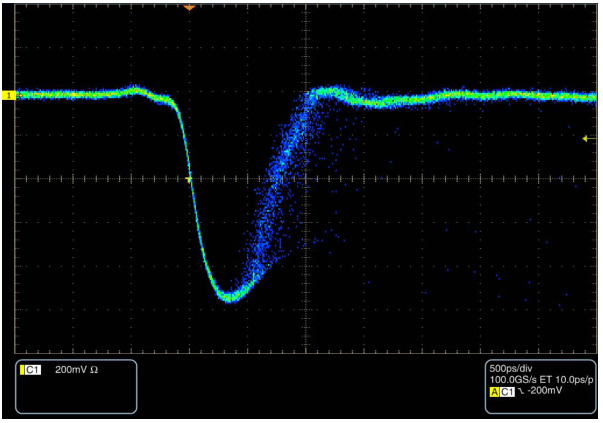
One of the two output voltage signals, $v_{\mathrm{out}}$, captured using a 4-GHz bandwidth oscilloscope (TDS7404B, Tektronix, Inc., Beaverton, OR, USA) under moderate illumination and displayed with low persistence to highlight its typical shape. The pulse exhibits a negative width of ≈750 ps FWHM, with an effective low-threshold duration of ≈1 ns.

The key performance metrics of the developed detection module are the effective system dead time and the Instrument Response Function (IRF). Together, these parameters provide a consistent framework to quantify the fundamental trade-off between high-count-rate capability and optimal timing jitter, as determined by the selected SiPM combined with the overall front-end architecture. Figure 5 shows one of the two output signals, namely the negative voltage pulse, $v_{\mathrm{out}}$, captured with a 4-GHz bandwidth oscilloscope (TDS7404B, Tektronix, Inc., Beaverton, OR, USA) under moderate illumination and displayed with low persistence to highlight its typical shape. The recorded waveform exhibits exceptionally clean transitions, with a measured fall time of ≈270 ps, a rise time of ≈500 ps, and a pulse width of ≈750 ps FWHM. The effective low-threshold duration—evaluated both before and after the comparator stage—is ≈1 ns.

**Figure 6:**
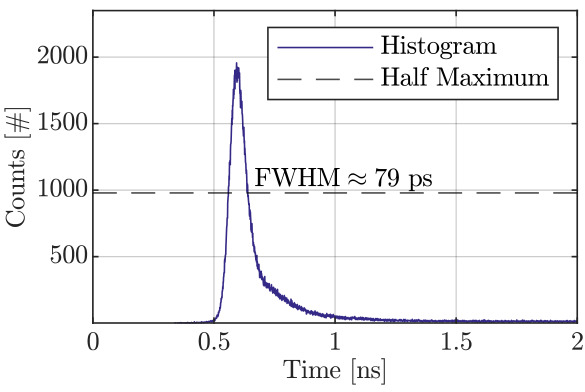
Detection module IRF with pulse width of ≈79 ps FWHM. The y-axis shows the number of counts in the resulting histogram.

The IRF is reported in Figure 6. It was measured using a pulsed laser diode (MPL-820, Antel Optronics Inc.) emitting at 820nm with a pulse width of approximately 10psFWHM. The detected response is acquired using a commercial TCSPC module (SPC-130, Becker & Hickl GmbH, Berlin, Germany) hosted in a PC, under the same voltage bias conditions reported in Section 2. Both the laser and the event timer contribute negligibly to the overall timing jitter. The photon count rate was kept below 5% of the excitation frequency, set to 100kHz, to avoid pile-up distortion. The measured IRF exhibits a dominant peak with a ≈79 ps FWHM—corresponding to ≈33.5 ps RMS—followed by an exponential decaying tail. At a wavelength of 820nm, a fraction of the incident photons is absorbed in the substrate rather than directly within the high-field depletion region. The corresponding photogenerated carriers must then diffuse into the active region before triggering the avalanche process, resulting in a delayed response that manifests as the long tail following the main peak [26]. Similar results have been observed in [46]. The dominant timing jitter contribution of the measured IRF can reasonably be attributed to residual on-board circuitry, layout-related effects and external noise sources, since the contribution from the readout, amplification, and comparator stages is limited by the equivalent input rise-time bandwidth of the comparator, and the SiPM device is expected to introduce only negligible additional effects related to transient time delays between distinct micro-cells [47] or to SPAD-to-SPAD variations—likely due to reduced electric field border-related non-uniformities [25,34,46].

All measured metrics show good agreement with the simulated results reported in Section 4, with minor deviations mainly attributable to slight variations in the SiPM model parameters and the tolerances of the front-end components. Overall, the optimized front-end enables reliable operation at high count rates while preserving excellent temporal resolution, establishing the system as a highly effective solution for ultra-fast fluorescence measurements aimed at overcoming the intrinsic limitations of conventional TCSPC architectures.

## 5. Fluorescence Measurements

In this study, we demonstrate the significant advantages of the SiPM-based detection module for fluorescence measurements. The accurate measurement of the lifetime decay is ensured by the recently developed constraint-free TCSPC methodology mentioned in Section 1. First, we briefly review the main principles of the proposed TCSPC measurement strategy [16,17,18].

### 5.1. Pile-Up Correction Strategy

We define the laser period, during which the sample is repetitively excited, as $\left[0,\;T_{\mathrm{laser}}\right]$. The function $r_{\mathrm{rec}}\left(t\right)$ represents the distribution of recorded photons within $\left[0,\;T_{\mathrm{laser}}\right]$, affected by the dead time of the detection system. Indeed, each detected event momentarily disables the detection system, potentially introducing pile-up effects that distort the recorded data histogram and corrupt the actual fluorescence dynamics. In contrast, the function $r_{\mathrm{imp}}\left(t\right)$ represents the distribution of the impinging photons within the period, representing the uncorrupted fluorescence decay profile. The relationship that enables the reconstruction of $r_{\mathrm{imp}}\left(t\right)$ from $r_{\mathrm{rec}}\left(t\right)$, is given by

(9) $$\begin{array}{c} r_{\mathrm{imp}}\left(t\right)=\frac{r_{\mathrm{rec}}\left(t\right)}{\alpha (t)}, \end{array}$$

where α(t) represents the probability that the detection system is active and capable of successfully registering a photon event at a given time $t\in [0,\;T_{\mathrm{laser}}]$ during the measurement process. A detailed derivation of α(t) and its computation methodology for the SiPM-based module presented in this work are provided in Appendix A.

### 5.2. Experimental Setup

**Figure 7:**
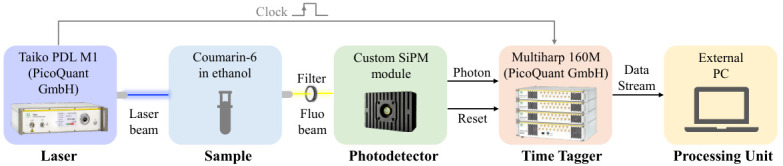
Experimental setup for fluorescence measurements. A pulsed laser excites a Coumarin-6 solid sample diluted in ethanol. The emitted fluorescence signal is attenuated through circular reflective neutral density filters prior to detection performed by the photodetector. The output voltage pulses are split and routed to two input channels of a commercial multichannel event timer, operating in time-tagging mode and synchronized with a reference clock signal provided by the laser. The data stream generated by the time tagger are subsequently processed by an external PC.

The TCSPC setup integrating the custom detection module is illustrated in Figure 7. The fluorescence sample consisted of a Coumarin-6 dye solid, diluted in ethanol. Excitation was provided by a high-performance picosecond diode laser (Taiko PDL M1, PicoQuant GmbH, Berlin, Germany), coupled to a cuboid laser head (LDH-IB-470-B, PicoQuant GmbH, Berlin, Germany). The system delivers laser pulses at a wavelength of 470nm with a pulse width of approximately 80psFWHM. Throughout the measurements, the detection system was actively cooled to 10 °C, which provides a stable and robust thermal operating point while ensuring a sufficiently low dark count rate for the operating conditions explored in this study. The negative output voltage pulse, illustrated in Figure 5, was subsequently split and routed to two independent input channels of a multichannel event timer (MultiHarp 160 M, PicoQuant GmbH, Berlin, Germany) operating in time-tagging mode. The time tagger was synchronized to a stable reference clock generated from the laser trigger signal. Each photon arrival time was acquired on one channel via the falling-edge detection associated with the front of the negative signal pulse, while the end of the photodetector dead time was registered on a second channel via the rising-edge detection associated with its decay. Experimental observations guided the selection of a threshold voltage of −200mV for the time tagger, providing optimal noise rejection and robust detection performance.

The laser repetition rate was set at 20MHz, to avoid temporal refolding artifacts and the saturation of the time tagger, ensuring correct operation within its maximum achievable throughput. Importantly, the following measurements were intentionally performed to reach multiple photons per excitation period, rather than to maximize the absolute count rate. The laser output power was fixed at 250μW. A series of neutral density filters (Thorlabs Inc., Newton, NJ, USA) with progressively decreasing attenuation factors was employed to systematically modulate the emitted photon flux while preserving the temporal characteristics of the excitation pulse. This approach enabled controlled variation in the average number of photons impinging on the photodetector per laser period, ensuring extensive exploration of pile-up effects under different illumination intensities. The raw timestamped photon data acquired by the event timer were transmitted to an external PC for post-processing. Fluorescence decay histograms were reconstructed with 10ps temporal resolution. Finally, in the complete measurement set, the IRF described in Section 4 is expected to play a minor role, being narrower at the sample emission wavelengths (500–550nm) than at 820nm, due to reduced diffusion effects. Similar observations are reported in [46,56].

### 5.3. Results and Discussion

**Figure 8:**
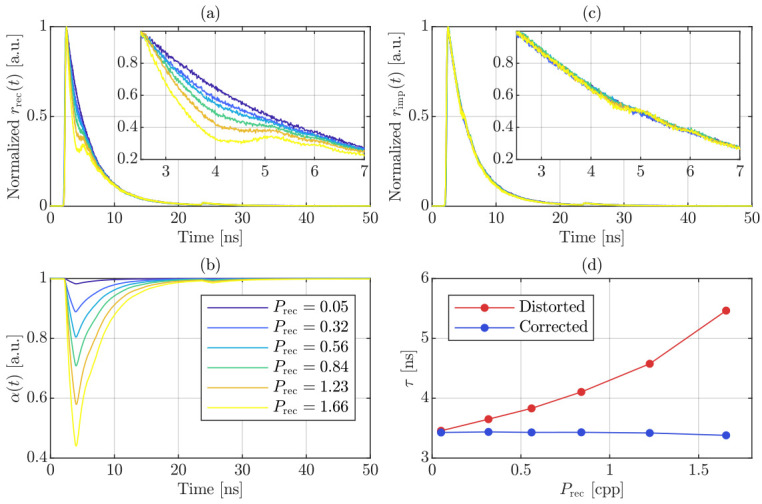
Application of the pile-up correction strategy as $P_{\mathrm{rec}}$ increases. (**a**) Normalized recorded data histograms, $r_{\mathrm{rec}}\left(t\right)$; (**b**) detection probability function, α(t), with legend in common to panels (**a**,**c**); and (**c**) normalized impinging data histograms, $r_{\mathrm{imp}}\left(t\right)$, after the application of Equation (9). (**d**) Estimated lifetime values from $r_{\mathrm{rec}}\left(t\right)$ in red and $r_{\mathrm{imp}}\left(t\right)$ in blue as a function of $P_{\mathrm{rec}}$.

The experimental findings are illustrated in Figure 8. In accordance with classical theoretical constraints, an initial TCSPC measurement was conducted, keeping the recorded photon count rate below 5% of the excitation frequency to minimize pile-up distortion. The resulting normalized fluorescence decay curve is visible in dark blue in Figure 8a and serves as a reliable reference. In particular, it shows a fluorescence lifetime, $\tau_{\mathrm{ref}}$, equal to 3.43ns, in agreement with the expected photophysical properties of the sample. As the average recorded photon count rate per period, $P_{\mathrm{rec}}$, increases toward 166% of the excitation frequency, corresponding to an average of 1.66 photons per laser pulse, pile-up artifacts become increasingly pronounced in the recorded data histogram, as highlighted in the zoomed-in inset of Figure 8a. In particular, the distortion manifests as a sharp dip immediately following the peak of the distribution, severely compromising the accuracy of direct lifetime estimation. Figure 8b shows the correction function, α(t), for progressively increasing $P_{\mathrm{rec}}$. The application of Equation (9) results in the corrected light profiles emitted by the sample, as shown in Figure 8c, effectively restoring the actual fluorescence dynamics even under extreme photon flux conditions. It is worth noting that, in the reported measurements, no significant degradation in data integrity attributable to false-photon events arising from afterpulsing or crosstalk was observed, due to the characteristics of the tailored SiPM employed, whose optimized design lead to intrinsically low correlated noise contributions under the investigated operating conditions.

Figure 8d reports the estimated fluorescence lifetime as a function of $P_{\mathrm{rec}}$. Specifically, lifetime estimation was performed employing a built-in Non-Linear Least Squares fitting function in MATLAB (R2024b, The MathWorks, Inc., Natick, MA, USA), applying a monoexponential decay model. The lifetime values extracted from the raw data histograms are shown in red, while the lifetime values derived from the corrected histograms are shown in blue. In the absence of the pile-up correction methodology, the recorded lifetime at 166% of the excitation rate yields an incorrect value of 5.47ns. However, after applying Equation (9), $r_{\mathrm{imp}}\left(t\right)$ accurately recovers the fluorescence lifetime emitted, reducing the estimated value to 3.38ns, closely matching $\tau_{\mathrm{ref}}$. Under this extremely challenging measurement condition, the percentage error deviation from the reference value is drastically reduced from an unacceptable +59.48% to a mere −1.46%.

**Figure 9:**
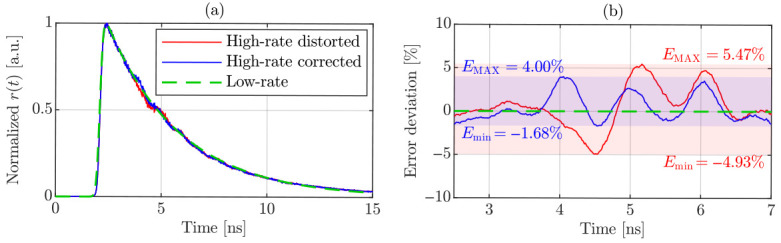
Application of the hardware-induced distortion correction algorithm combined with the pile-up compensation strategy. (**a**) Experimental histograms at $P_{\mathrm{rec}}=166\%$: distorted data in red and corrected data in blue after applying Equation (9). (**b**) Percentage error deviation with respect to the measured low-count-rate reference (dashed green curve), evaluated using a 30-sample moving average.

Although the fluorescence lifetimes extracted from the corrected histograms remain highly accurate over the entire range of investigated operating conditions, a subtle systematic distortion persists in the corrected distributions. This effect, highlighted in the magnified inset of Figure 8c, becomes particularly pronounced at the highest investigated recorded photon rate. In fact, while the proposed correction methodology successfully compensates for photon losses induced by the photodetector dead time, it does not completely suppress eventual secondary artifacts that arise in the region of the decay immediately afterward. The residual deformation observed in the yellow trace in the inset of Figure 8c therefore suggests the presence of higher-order system-level effects beyond conventional pile-up distortion superimposed on the ideal mono-exponential decay. In TCSPC-based fluorescence measurements, photon arrivals follow inhomogeneous Poisson statistics under repetitive excitation, with the detection probability peaking at the maximum of the fluorescence decay. Consequently, under high photon-flux conditions, multiple photons can be detected within the same excitation cycle, and the probability of detecting a subsequent photon immediately after the dead time imposed by a preceding event is maximized accordingly. Given the temporal shape of $v_{\mathrm{filter}}$, if a second photon arrives while the first pulse is still in its undershoot phase, it superimposes onto a non-zero residual baseline. This residual transient shifts the effective zero-crossing point at the comparator input, thereby introducing a deterministic temporal bias in the registered timestamp. Accounting for this effect leads to an average effective dead time, $T_{\mathrm{dead}}$, of 1.68ns, which exceeds the output pulse width measured at the low-threshold level. To further mitigate residual hardware-induced artifacts—particularly relevant for complex multi-exponential decay profiles—we applied a recently reported correction algorithm in addition to the pile-up correction methodology [18]. The proposed algorithm is based on the identification and compensation of the deterministic temporal bias introduced by the voltage undershoot following a photon-induced avalanche event. Its operating principle relies on experimentally characterizing the system response to photon pairs with a precisely controlled temporal delay, and it can be successfully applied under the specific measurement conditions presented in this study. Since the calibration procedure associated with the validation algorithm depends exclusively on the output pulse shape, it can, in principle, be carried out only once, assuming stable pulse-shape characteristics over time. Using the same experimental configuration and implementation procedure described in [18], we evaluated the performance of the proposed correction strategy in combination with the pile-up compensation approach. Figure 9a shows the histograms at the maximum recorded photon flux, while Figure 9b reports the percentage error deviation of both with respect to the low-count-rate reference distribution (dashed green curve), evaluated using a 30-sample moving average to improve clarity. The red curve corresponds to data corrected exclusively for pile-up, whereas the blue curve represents the histogram obtained by applying the temporal-shift correction algorithm prior to pile-up compensation. Under the same fitting conditions employed in Figure 8, the lifetime extracted for the blue curve in Figure 9a is slightly lower—by approximately 90 ps—than that obtained for the red curve, a small difference most plausibly attributed to the sensitivity of the fitting procedure rather than to any discrepancy introduced by the correction method. Instead, the peak-to-peak error deviation is reduced from 10.4% to 5.68%, thus partially compensating for $v_{\mathrm{filter},\mathrm{peak}}^{-}/v_{\mathrm{filter},\mathrm{peak}}^{+}\approx 47.6\%$, which was not explicitly prioritized during the optimization procedure, while further mitigating the residual distortion effects.

## 6. Conclusions

In this work, we presented a custom-designed SiPM-based detection module tailored for ultra-fast time-resolved TCSPC-based applications. Table 3 provides a comparative overview with other state-of-the-art single-channel SiPM-based detection systems reported in the literature for time-resolved measurements, to the best of our knowledge. The comparison intentionally refers to fully implemented systems, rather than to isolated performance metrics. As reflected by the results, the proposed module achieves an ideal balance between minimal system dead time and high-precision photon timing, offering a robust and efficient solution that advances the capabilities of TCSPC, particularly under high-rate conditions. Experimental results in fluorescence measurements demonstrate an exceptional photon count rate of 166% of the excitation frequency combined with a peak-to-peak lifetime estimation error ≤1.46% across all measurements. Additionally, the employment of the recently reported algorithm effectively mitigates hardware-induced residual distortions, enabling high-fidelity optical waveform reconstruction, even in the most photon-congested acquisition regimes. By integrating an optimized front-end architecture with a carefully engineered signal-processing strategy, the proposed system establishes a new benchmark for detection modules in advanced single-photon timing applications, where ultra-fast data acquisition and processing are essential and the highest possible precision is required.

Future efforts will concentrate on integrating both the hardware correction algorithm and the pile-up correction methodology onto an on-board FPGA. This approach will enable near-real-time processing, ensuring faster and more accurate corrections while significantly improving system integration.

## Figures and Tables

**Table 1:** SiPM simulation model component parameters.

Parameter	Value	Parameter	Value
*N*	4830	$C_{q}$	4 fF
$R_{s}$	800 Ω	$C_{g}$	5 pF
$R_{q}$	500 kΩ	$L_{b}$	4 nH
$C_{d}$	8 fF		

**Table 2:** Front-end component parameters.

Parameter	Value	Parameter	Value
$R_{\mathrm{sense}}$	39 Ω	$R_{T,1},R_{T,2}$	330 Ω
$R_{\mathrm{in},\mathrm{OA}}$	100 Ω	$R_{B,1},R_{B,2}$	33 Ω
$R_{\mathrm{out},\mathrm{OA}}$	7 Ω	$R_{E}$	220 Ω
$R_{1},R_{3}$	43 Ω	$C_{\mathrm{cpl}}$	820 pF
$R_{\mathrm{shunt}}$	82 Ω	$C_{1}$	1 nF
$R_{\mathrm{th},1},R_{\mathrm{th},2}$	1.2 kΩ	$C_{2}$	20 pF
$R_{\mathrm{in},\mathrm{COMP}}$	100 Ω		

**Table 3:** Comparison of single-channel SiPM detection modules for time-resolved applications.

	This Work	Acerbi et al. (2021)	Martinenghi et al. (2016)
Year—Reference	2026	2021—[57]	2016—[22]
$A_{\mathrm{SiPM}}$ [mm × mm]	1 × 1	6 × 6	1 × 1
Cell pitch [μm]	15	35	50
Fill factor [%]	63.6	>80	51
$V_{\mathrm{BD}}$ @ 25 °C [V]	32	33	95.6
IRF [ps FWHM (nm)]	79	550–720	100
	(@ 820)	(@ 600–1100)	(@ 600–1000)
Output pulse width [ns FWHM]	0.75	10 ^1^	NA
Module power consumption [W]	1.8	0.9	6 ^2^
Module size [cm × cm × cm]	10 × 7 × 3.7	5 × 5 × 4	5 × 4 × 10

^1^ Information extrapolated from the reference paper but not directly reported. ^2^ Power consumption includes the TEC controller.

## Data Availability

The raw data supporting the conclusions of this article will be made available by the authors on request.

## Appendix A. Correction Function Derivation

The correction function derivation is detailed in this Appendix. α(t) is constructed at run-time through the continuous monitoring of the operational state of the detection system and serves as a time-dependent correction factor for the recorded data histogram to compensate for distortions arising from inevitable dead-time effects [16]. Specifically, upon photon detection, the system enters dead time, $T_{\mathrm{dead}}$, during which it is temporarily unable to register additional photons. After this interval, it regains the sensitivity to record eventual subsequent events. The excitation period is discretized into finite time bins according to the temporal resolution of the processing electronics. High-resolution timestamps are assigned to photon detection events and their corresponding reset signals, allowing the count in each time bin of the correction histogram to be updated based on the instantaneous efficiency of the photodetector. Considering $N_{f}\left(t\right)$ as the number of micro-cells fired at *t* $\in [0,\;T_{\mathrm{laser}}]$, this time-dependent efficiency is quantified as follows:

(A1) $$\begin{array}{c} \varepsilon \left(t\right)=1-\frac{N_{f}\left(t\right)}{N}. \end{array}$$

A critical distinction must be made between the macroscopic ability of the system to detect photons and the microscopic recovery dynamics of individual micro-cells. Each one, upon detecting a photon, becomes temporarily inactive and requires a finite recovery time before its detection capability is fully restored. As a result, the overall detection efficiency of the SiPM is dynamically modulated by the collective recovery status of its micro-cells described by Equation (A1). The greater the number of cells still recovering, the lower the probability that an incoming photon will be registered. During $T_{\mathrm{dead}}$, the SiPM front-end electronics inhibit any further event registration even if ε(t)≠0 and the system is completely insensitive to new photon arrivals. In this interval, α(t)=0 independently of ε(t). Once $T_{\mathrm{dead}}$ elapsed, ε(t) evolves according to Equation (A1), and the corresponding bins of α(t) should be incremented proportionally to the fraction of micro-cells available to detect a new event, i.e., α(t)=ε(t). Nevertheless, for the specific SiPM employed in this study, *N* is quite high and, at the photon flux levels investigated in this work, the number of micro-cells fired at any given time instant remains negligible compared to the total cell count. As a result, the reduction in detection efficiency due to micro-cell saturation is minimal, and the per-bin increment in α(t) can be reasonably approximated as unity without introducing a significant error, i.e., α(t)=ε(t)≈1. This reconstruction procedure is repeated across all excitation cycles. Once completed, a normalization step is applied with respect to the total number of measurement periods, ensuring that α(t) is constrained within the interval [0,1]. Each bin of α(t) then represents the probability that the photodetector was in an active state at the corresponding time instant $t\in [0,\;T_{\mathrm{laser}}]$.

## References

[1] Becker W. (2015). Advanced Time-Correlated Single Photon Counting Applications.

[2] Hirvonen L.M., Suhling K. (2016). Wide-field TCSPC: Methods and applications. Meas. Sci. Technol..

[3] Dutton N.A., Gnecchi S., Parmesan L., Holmes A.J., Rae B., Grant L.A., Henderson R.K. (2015). 11.5 A time-correlated single-photon-counting sensor with 14GS/S histogramming time-to-digital converter. Proceedings of the 2015 IEEE International Solid-State Circuits Conference-(ISSCC) Digest of Technical Papers.

[4] Acconcia G., Malanga F., Farina S., Ghioni M., Rech I. (2023). A 1.9 ps-rms Precision Time-to-Amplitude Converter with 782 fs LSB and 0.79%-rms DNL. IEEE Trans. Instrum. Meas..

[5] Köllner M., Wolfrum J. (1992). How many photons are necessary for fluorescence-lifetime measurements?. Chem. Phys. Lett..

[6] Suhling K., Hirvonen L.M., Levitt J.A., Chung P.H., Tregidgo C., Le Marois A., Rusakov D.A., Zheng K., Ameer-Beg S., Poland S. (2015). Fluorescence lifetime imaging (FLIM): Basic concepts and some recent developments. Med. Photonics.

[7] Liu X., Lin D., Becker W., Niu J., Yu B., Liu L., Qu J. (2019). Fast fluorescence lifetime imaging techniques: A review on challenge and development. J. Innov. Opt. Health Sci..

[8] Datta R., Heaster T.M., Sharick J.T., Gillette A.A., Skala M.C. (2020). Fluorescence lifetime imaging microscopy: Fundamentals and advances in instrumentation, analysis, and applications. J. Biomed. Opt..

[9] Rech I., Luo G., Ghioni M., Yang H., Xie X.S., Cova S. (2004). Photon-timing detector module for single-molecule spectroscopy with 60-ps resolution. IEEE J. Sel. Top. Quantum Electron..

[10] Tobin R., Halimi A., McCarthy A., Soan P.J., Buller G.S. (2021). Robust real-time 3D imaging of moving scenes through atmospheric obscurant using single-photon LiDAR. Sci. Rep..

[11] Maccarone A., Acconcia G., Steinlehner U., Labanca I., Newborough D., Rech I., Buller G.S. (2021). Custom-technology single-photon avalanche diode linear detector array for underwater depth imaging. Sensors.

[12] Arlt J., Tyndall D., Rae B.R., Li D.D.U., Richardson J.A., Henderson R.K. (2013). A study of pile-up in integrated time-correlated single photon counting systems. Rev. Sci. Instrum..

[13] Zhou X., Bec J., Ehrlich K., Garcia A.A., Marcu L. (2023). Pulse-sampling fluorescence lifetime imaging: Evaluation of photon economy. Opt. Lett..

[14] Walker J.G. (2002). Iterative correction for ’pile-up’ in single-photon lifetime measurement. Opt. Commun..

[15] Isbaner S., Karedla N., Ruhlandt D., Stein S.C., Chizhik A., Gregor I., Enderlein J. (2016). Dead-time correction of fluorescence lifetime measurements and fluorescence lifetime imaging. Opt. Express.

[16] Rech I., Bovolenta A., Cominelli A., Acconcia G. (2023). Toward Constraintless Time-Correlated Single-Photon Counting Measurements: A New Method to Remove Pile-Up Distortion. IEEE J. Sel. Top. Quantum Electron..

[17] Fratta G., Daniele P., Labanca I., Acconcia G., Rech I. (2024). Near-zero distortion in TCSPC at more than one photon per excitation period: Experimental validation. Opt. Lett..

[18] Daniele P., Fratta G., Labanca I., Acconcia G., Rech I. (2025). Breaking boundaries of hybrid photodetector: A novel approach for high-speed TCSPC with minimal distortion. APL Photonics.

[19] Villa F., Markovic B., Bellisai S., Bronzi D., Tosi A., Zappa F., Tisa S., Durini D., Weyers S., Paschen U. (2012). SPAD smart pixel for time-of-flight and time-correlated single-photon counting measurements. IEEE Photonics J..

[20] Gramuglia F., Wu M.L., Bruschini C., Lee M.J., Charbon E. (2021). A low-noise CMOS SPAD pixel with 12.1 ps SPTR and 3 ns dead time. IEEE J. Sel. Top. Quantum Electron..

[21] Gulinatti A., Ceccarelli F., Ghioni M., Rech I. (2021). Custom silicon technology for SPAD-arrays with red-enhanced sensitivity and low timing jitter. Opt. Express.

[22] Martinenghi E., Di Sieno L., Contini D., Sanzaro M., Pifferi A., Dalla Mora A. (2016). Time-resolved single-photon detection module based on silicon photomultiplier: A novel building block for time-correlated measurement systems. Rev. Sci. Instrum..

[23] Caccia M., Nardo L., Santoro R., Schaffhauser D. (2019). Silicon Photomultipliers and SPAD imagers in biophotonics: Advances and perspectives. Nucl. Instrum. Methods Phys. Res. Sect. A Accel. Spectrometers Detect. Assoc. Equip..

[24] Michalet X., Cheng A., Antelman J., Suyama M., Arisaka K., Weiss S. (2008). Hybrid photodetector for single-molecule spectroscopy and microscopy. Proceedings of the Single Molecule Spectroscopy and Imaging.

[25] Acerbi F., Gundacker S. (2019). Understanding and simulating SiPMs. Nucl. Instrum. Methods Phys. Res. Sect. A Accel. Spectrometers Detect. Assoc. Equip..

[26] Piemonte C., Gola A. (2019). Overview on the main parameters and technology of modern Silicon Photomultipliers. Nucl. Instrum. Methods Phys. Res. Sect. A Accel. Spectrometers Detect. Assoc. Equip..

[27] Buzhan P., Dolgoshein B., Filatov L., Ilyin A., Kantzerov V., Kaplin V., Karakash A., Kayumov F., Klemin S., Popova E. (2003). Silicon photomultiplier and its possible applications. Nucl. Instrum. Methods Phys. Res. Sect. A Accel. Spectrometers Detect. Assoc. Equip..

[28] Golovin V., Saveliev V. (2004). Novel type of avalanche photodetector with Geiger mode operation. Nucl. Instrum. Methods Phys. Res. Sect. A Accel. Spectrometers Detect. Assoc. Equip..

[29] Renker D. (2006). Geiger-mode avalanche photodiodes, history, properties and problems. Nucl. Instrum. Methods Phys. Res. Sect. A Accel. Spectrometers Detect. Assoc. Equip..

[30] Herbert D.J., Saveliev V., Belcari N., D’Ascenzo N., Del Guerra A., Golovin A. (2006). First results of scintillator readout with silicon photomultiplier. IEEE Trans. Nucl. Sci..

[31] Renker D., Lorenz E. (2009). Advances in solid state photon detectors. J. Instrum..

[32] Acerbi F., Paternoster G., Gola A., Regazzoni V., Zorzi N., Piemonte C. (2018). High-density silicon photomultipliers: Performance and linearity evaluation for high efficiency and dynamic-range applications. IEEE J. Quantum Electron..

[33] Rech I., Marangoni S., Resnati D., Ghioni M., Cova S. (2009). Multipixel single-photon avalanche diode array for parallel photon counting applications. J. Mod. Opt..

[34] Gola A., Acerbi F., Capasso M., Marcante M., Mazzi A., Paternoster G., Piemonte C., Regazzoni V., Zorzi N. (2019). NUV-sensitive silicon photomultiplier technologies developed at Fondazione Bruno Kessler. Sensors.

[35] Corsi F., Marzocca C., Perrotta A., Dragone A., Foresta M., Del Guerra A., Marcatili S., Llosa G., Collazzuol G., Dalla Betta G.F. (2006). Electrical characterization of silicon photo-multiplier detectors for optimal front-end design. Proceedings of the 2006 IEEE Nuclear Science Symposium Conference Record.

[36] Corsi F., Dragone A., Marzocca C., Del Guerra A., Delizia P., Dinu N., Piemonte C., Boscardin M., Dalla Betta G.F. (2007). Modelling a silicon photomultiplier (SiPM) as a signal source for optimum front-end design. Nucl. Instrum. Methods Phys. Res. Sect. A Accel. Spectrometers Detect. Assoc. Equip..

[37] Seifert S., Van Dam H.T., Huizenga J., Vinke R., Dendooven P., Lohner H., Schaart D.R. (2009). Simulation of silicon photomultiplier signals. IEEE Trans. Nucl. Sci..

[38] Marano D., Bonanno G., Belluso M., Billotta S., Grillo A., Garozzo S., Romeo G., Catalano O., La Rosa G., Sottile G. (2013). Improved SPICE electrical model of silicon photomultipliers. Nucl. Instrum. Methods Phys. Res. Sect. A Accel. Spectrometers Detect. Assoc. Equip..

[39] Marano D., Belluso M., Bonanno G., Billotta S., Grillo A., Garozzo S., Romeo G., Catalano O., La Rosa G., Sottile G. (2013). Silicon photomultipliers electrical model extensive analytical analysis. IEEE Trans. Nucl. Sci..

[40] Villa F., Zou Y., Dalla Mora A., Tosi A., Zappa F. (2015). SPICE electrical models and simulations of silicon photomultipliers. IEEE Trans. Nucl. Sci..

[41] Gundacker S., Heering A. (2020). The silicon photomultiplier: Fundamentals and applications of a modern solid-state photon detector. Phys. Med. Biol..

[42] Ciciriello F., Corsi F., Licciulli F., Marzocca C., Matarrese G. (2014). Interfacing a SiPM to a Current-mode Front-end: Effects of the Coupling Inductance. Proceedings of the 2014 IEEE Nuclear Science Symposium and Medical Imaging Conference (NSS/MIC).

[43] Cova S., Ghioni M., Lacaita A., Samori C., Zappa F. (1996). Avalanche photodiodes and quenching circuits for single-photon detection. Appl. Opt..

[44] Calò P.P., Ciciriello F., Petrignani S., Marzocca C. (2019). SiPM readout electronics. Nucl. Instrum. Methods Phys. Res. Sect. A Accel. Spectrometers Detect. Assoc. Equip..

[45] Gola A., Piemonte C., Tarolli A. (2013). Analog circuit for timing measurements with large area SiPMs coupled to LYSO crystals. IEEE Trans. Nucl. Sci..

[46] Acerbi F., Ferri A., Gola A., Cazzanelli M., Pavesi L., Zorzi N., Piemonte C. (2014). Characterization of single-photon time resolution: From single SPAD to silicon photomultiplier. IEEE Trans. Nucl. Sci..

[47] Nemallapudi M.V., Gundacker S., Lecoq P., Auffray E. (2016). Single photon time resolution of state of the art SiPMs. J. Instrum..

[48] Gundacker S., Martinez Turtos R., Kratochwil N., Pots R.H., Paganoni M., Lecoq P., Auffray E. (2020). Experimental time resolution limits of modern SiPMs and TOF-PET detectors exploring different scintillators and Cherenkov emission. Phys. Med. Biol..

[49] Huizenga J., Seifert S., Schreuder F., Van Dam H., Dendooven P., Löhner H., Vinke R., Schaart D. (2012). A fast preamplifier concept for SiPM-based time-of-flight PET detectors. Nucl. Instrum. Methods Phys. Res. Sect. A Accel. Spectrometers Detect. Assoc. Equip..

[50] Fernandez-Tenllado J., Ballabriga R., Campbell M., Gascon D., Gomez S., Mauricio J. (2019). Optimal design of single-photon sensor front-end electronics for fast-timing applications. Proceedings of the 2019 IEEE Nuclear Science Symposium and Medical Imaging Conference (NSS/MIC).

[51] Zhang N., Schmand M.J. (2018). Bootstrapping Readout for Large Terminal Capacitance Analog-SiPM Based Time-of-Flight PET Detector. U.S. Patent.

[52] Cates J.W., Gundacker S., Auffray E., Lecoq P., Levin C.S. (2018). Improved single photon time resolution for analog SiPMs with front end readout that reduces influence of electronic noise. Phys. Med. Biol..

[53] Gundacker S., Turtos R.M., Auffray E., Paganoni M., Lecoq P. (2019). High-frequency SiPM readout advances measured coincidence time resolution limits in TOF-PET. Phys. Med. Biol..

[54] Frach T., Prescher G., Degenhardt C., De Gruyter R., Schmitz A., Ballizany R. (2009). The digital silicon photomultiplier—Principle of operation and intrinsic detector performance. Proceedings of the 2009 IEEE Nuclear Science Symposium Conference Record (NSS/MIC).

[55] Liu Z., Gundacker S., Pizzichemi M., Ghezzi A., Auffray E., Lecoq P., Paganoni M. (2016). In-depth study of single photon time resolution for the Philips digital silicon photomultiplier. J. Instrum..

[56] Puill V., Bazin C., Breton D., Burmistrov L., Chaumat V., Dinu N., Maalmi J., Vagnucci J., Stocchi A. (2012). Single photoelectron timing resolution of SiPM as a function of the bias voltage, the wavelength and the temperature. Nucl. Instrum. Methods Phys. Res. Sect. A Accel. Spectrometers Detect. Assoc. Equip..

[57] Acerbi F., Behera A., Dalla Mora A., Di Sieno L., Gola A. (2021). Single-photon detection module based on large-area silicon photomultipliers for time-domain diffuse optics. Instruments.

